# Persisting cognitive impairment predicts functional dependence at 1 year after stroke and transient ischemic attack: a longitudinal, cohort study

**DOI:** 10.1186/s12877-022-03609-z

**Published:** 2022-12-31

**Authors:** Xiaoling Liao, Lijun Zuo, Yanhong Dong, Yuesong Pan, Hongyi Yan, Xia Meng, Hao Li, Xingquan Zhao, Yilong Wang, Jiong Shi, Yongjun Wang

**Affiliations:** 1 Department of Neurology, Beijing Tiantan Hospital, Capital Medical University, No.119, South Fourth Ring West Road, Fengtai District, Beijing, 100070 China; 2 Alice Lee Centre for Nursing Studies, Yong Loo Lin School of Medicine, National University of Singapore, Clinical Research Centre, Block MD11, Level 2, 10 Medical Dr., Singapore, 117597 Singapore; 3 National Clinical Research Center for Neurological Diseases, Beijing Tiantan Hospital, Capital Medical University, Beijing, China

**Keywords:** Mild stroke, Persisting cognitive impairment, Montreal cognitive assessment-Beijing, Functional dependence

## Abstract

**Objective:**

Minor stroke or transient ischemic attack (TIA) usually have mild and nondisabling symptoms, and these functional deficits may recover fully e.g., TIA, however, part of them still suffer from cognitive impairment and poor outcomes. We conducted a study to determine the relationship between cognition evaluated by Montreal Cognitive Assessment (MoCA) and poor functional outcomes assessed by the Modified Rankin Scale (mRS) (mRS ≥ 2) and Stroke Impact Scale (SIS)-16(SIS-16<25%).

**Methods:**

The data of this study come from the impairment of cognition and Sleep (ICONS) after acute ischemic stroke or transient ischemic attack in Chinese patients study. A total of 1675 minor stroke patients and TIA patients were finally recruited. Patients’ cognition were evaluated by Montreal Cognitive Assessment (MoCA) scale at 2-week (2w), 3 months (3 m) and 1 year(1y). Cognitive impairment (CI) was defined as MoCA score ≤ 22. According to MoCA score, patients were divided into 4 groups: no PSCI group: with MoCA-2w>22 and MoCA-3 m>22; improved PSCI group: with MoCA-2w ≤ 2 and MoCA-3 m>22;delayed PSCI group: MoCA-2w>22 and MoCA-3 m ≤ 22; persisting PSCI group: with MoCA-2w ≤ 22 and MoCA-3 m ≤ 22.

**Results:**

A total of 1675 stroke patients were recruited in this study. There were 818 patients (48.84%) who had PSCI at baseline. Of these, 123 patients (15%) had mRS ≥2 at 3 months. The persisting PSCI group was a significant predictor of functional dependence at 3 months and 1 year after stroke and when adjusted for covariates such as gender, age, history of stroke, depression and intracranial atherosclerotic stenosis, stroke subtype and acute infarction type.

**Conclusion:**

Persisting PSCI increased the risk of poor functional outcome after 3 months and 1 year follow-up. These high-risk individuals should be identified for targeted rehabilitation and counseling to improve longer-term post-stroke outcome.

**Supplementary Information:**

The online version contains supplementary material available at 10.1186/s12877-022-03609-z.

## Introduction

Patients with minor stroke have mild symptoms and may go through a rapid physical recovery. However, they may struggle with more complex activities and experience cognitive impairment. Studies have reported rates of cognitive impairment ranging from 35 to 92% according to different evaluation time and scales [1]. Impairment has been reported to affect multiple cognitive domains [2], one recent study evaluating those with TIA and minor stroke found difficulty with executive function and psychomotor processing to be the most common cognitive deficits [3]. The widely used screening tool is Mini-Mental State Examination (MMSE) and MoCA. MoCA includes executive function and attention tests (among many different cognitive domains), which have been more suitable thus recommended for screening for cognitive impairment in patients with stroke or TIA [4]. Therefore, we used MoCA to evaluate cognitive status in the present study. The effects and outcomes of stroke can be devastating. The mRS score is commonly used to assess functional outcomes, but it has ceiling effect. The Stroke Impact Scale (SIS)-16 was designed to comprehensively assess stroke-related outcomes [5] and accurately assess recovery after stroke [6]. It contains 8 domains – strength, hand function, activities of daily living, mobility, communication, emotion, memory and thinking, participation [6]. It has been applied to evaluate the health-related quality of life in post-stroke patients [7], on discharge and at the first and the third-month post-stroke [6]. Previous studies investigated the relationship between cognitive decline and function outcomes after stroke [8–10]. Early screening through MoCA could predict long-term functional dependence [11]. Higher baseline scores and greater improvement of cognition were significantly associated with lower mortality at 1 year after stroke [12]. Cognitive tests could predict functional outcomes, including daily activities, return to work and driving. Neuropsychological assessment at acute stroke phase could predict functioning at work and fulfilling social roles at 1-year post-stroke [13, 14]. However, previous studies have primarily been cross-sectional, rather than longitudinal. The MoCA was administered within 7 days to > 1 year after stroke [9, 15] with just one timepoint. The studies also had a small sample size [9, 16]. Acute temporary cognitive deficits after minor stroke/TIA are common, and these cognitive deficits may recover to some extent (transient cognitive impairment [TCI]) over time, and are not in line with physical recovery [17]. About 57% patients with TIA/minor stroke have one or more impaired neuropsychological tests within 1 week [18] compared to only 19% of those seen after 7 days [17]. There are both transient and long-term cognitive impairment after TIA [19]. The changes of cognitive impairment after TIA/minor stroke may be improved, stable or declined. The executive dysfunction is a prominent feature after TIA [20]. No previous study has used serial assessment to examine the temporal profile of cognitive impairments at 2 week(2w), 3 month(3 m) and 1 year(1y) after TIA/minor stroke. Additionally, there is no study investigating the association between different cognitive change patterns and functional outcomes after stroke at 1-year follow-up.

A MoCA-Beijing ≤22 has been defined as cognitive impairment at 2 weeks after minor TIA/stroke [21]. In this study, we investigate the relationship between the different cognitive change patterns and 1-year functional outcomes.

## Methods and materials

### Subjects

The present cohort was from the Impairment of CognitiON and Sleep after acute ischemic stroke or transient ischemic attack in Chinese patients (ICONS) study. ICONS is one of the research subgroups of China National Stroke Registry-III (CNSR-III), which is a nationwide prospective registry for patients presented to hospitals with AIS or TIA between August 2015 and March 2018 from 201 hospitals that covers 22 provinces and four municipalities in China. The detailed design, rationale, and basic description of the CNSR-III have been published previously [22]. We recruited only minor stroke in this study. The minor stroke was defined as the National Institutes of Health Stroke Scale (NIHSS) [23] score ≤ 3. Cognitive function was evaluated by MoCA at 2-week (2w), 3-month (3 m), and 1 year(1y) after TIA/minor stroke in Chinese patients. Functional outcomes were evaluated by Modified Rankin Scale (MRS) and Stroke Impact Scale (SIS-16) at 3-month (3 m) and 1 year(1y).

**Fig. 1:**
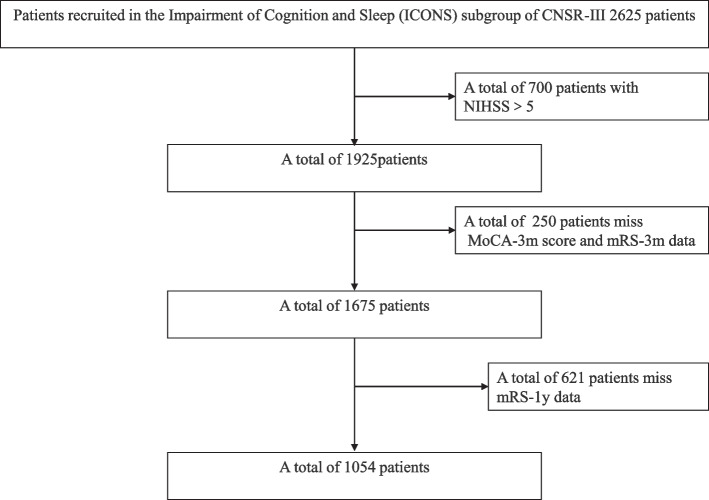
Flowchart of patients participating in the study

We excluded the patients who have stroke mimics (ie, seizures, migraine), illiteracy, history of dementia, aphasia, hemispatial neglect, disturbance of consciousness or limb dyskinesia and any major mental conditions that may impede cognitive assessments. Total 2625 patients enrolled in ICONS subgroup with MoCA-2w. We excluded 700 patients with NIHSS>5, and 250 patients without MoCA-3 m score and mRS-3 m data. Finally, 1675 patients of TIA/minor stroke completed MoCA-2w and MoCA-3 m tests, as well as mRS-3 m (Fig. 1). And then, we excluded 621 patients without mRS-1y data. There were 1054 patients with TIA/minor stroke completed MoCA-2w and MoCA-3 m tests, as well as mRS-3 m and mRS-1y (Fig. 1).

### Data collection

All study investigators were trained and certified to assess NIHSS scores before the beginning of the study. We collected baseline information including patient demographics, vascular risk factors, stroke severity (NIHSS score), stroke management discharge and drugs status. Vascular risk factors included hypertension, diabetes, lipid metabolism disorders, atrial fibrillation, previous stroke or TIA, current or previous smoking and body mass index (BMI) at admission. Etiologic subtypes of ischemic stroke were classified by the Stop Stroke Study Trial of Org 10,172 in Acute Stroke Treatment (SSS-TOAST) classification criteria. In ICONS study, MRI were recommended for all patients, including Diffusion-Weighted Imaging (DWI) with Apparent Diffusion Coefficient (ADC) maps, T1 weighted, T2 weighted, Fluid-attenuated Inversion Recovery (FLAIR), T2*/Susceptibility Weighted Imaging (SWI), and Magnetic Resonance Angiography (MRA). Acute infarction type including single infarction, multiple infarctions, simple watershed infarction and no infarction, and were completed by Imaging experts.

At 2-week or discharge, 3-month and 1-year, MoCA-Beijing [24]，Pittsburgh Sleep Quality Index (PSQI) [25], Epworth Sleeping Scale(ESS) [26], Anxiety Disorder-7(GAD-7) [27], and Patient Health Questionnaire-9(PHQ-9) [28] were evaluated face to face [5]. The detailed design, rationale, and basic description of the ICONS have been published previously [29]. All tests above were administered by trained examiners. In the medication survey at each follow-up point, information about whether combined with dual antiplatelet therapy and intravenous thrombolysis after stroke onset was collected.

### Outcome assessment

The follow-up was done by face-to-face interview. Patients were asked the standardized follow-up questions at 3 months and 1 year after stroke onset. Outcome data included the scores of mRS and SIS-16. The poor functional outcome was defined as a score of 2 to 6 on the mRS [30] . Poor physical and social functioning was defined as a percentage of SIS-16<25% [31].

### Diagnosis of VCI

Patients in this study with MoCA-Beijing ≤22 defined as cognitive impairment after TIA/minor stroke according to the results of our previous study recruited 102 patients after TIA/minor stroke at 2 weeks in China with MoCA-Beijing test and a formal neuropsychological test battery [21]. Our previous data showed that the optimal cutoff point for MoCA-Beijing in discriminating patients with CI from those with no cognitive impairment (NCI) was 22/23 (sensitivity 85%, specificity 88%, PPV = 91%, NPV = 80%). According to the results of cognitive evaluation, we divided patients into 4 groups as mentioned above: A group (no PSCI group): with MoCA-2w >22 and MoCA-3 m>22; B group(improved PSCI group): with MoCA-2w ≤ 22 and MoCA-3 m>22; C group (delayed PSCI group): MoCA-2w >22 and MoCA-3 m ≤ 22; D group (persisting PSCI group): with MoCA-2w ≤ 22 and MoCA-3 m ≤ 22.

### Statistical analyses

All statistical analyses were carried out with SAS 9.4 software (SAS Institute Inc., Cary, NC). The differences in baseline demographic and clinical features between NCI and CI were tested for continuous variables with normal distribution using Student-t test and with skewed distribution using nonparametric test. The χ^2^ or Fisher exact test was used for categorical variables. We analyzed the association between the clinical outcomes including early recurrent stroke, stroke disability and all-cause death and relevant covariates with logistic regression analysis adjusting age, gender, previous stroke, TOAST subtype, acute infarction type, and dual antiplatelet therapy after stroke onset. We have determined that two-tailed *p* values less than 0.05 was statistically significant.

## Results

### Baseline characteristics of TIA/minor stroke patients

Among the 2625 patients in the ICONS database, a total of 1675 had completed MoCA-2w, MoCA-3 m tests and mRS-3 m, the baseline and clinical features of the included 1675 patients are demonstrated in Table 1. Overall, the subjects with cognitive impairment (MoCA≤22) accounted for 48.84% of the total studied population at 2 weeks after stroke. Patients were divided into cognitive impairment (CI) group and non-cognitive impairment(NCI) group. The CI group were more likely to be elderly and female. They were also more likely to have a history of stroke, combination of sleep disorders(PSQI>5), depression(PHQ-9>9), and intracranial atherosclerotic stenosis. They have a higher percentage of receiving dual antiplatelet medications after onset. The acute infarction type and stroke etiology was imbalanced between the two groups. No significance was detected in the treatment for hypertension, diabetes mellitus, use of antiplatelet agents and statin.

**Table 1:** Comparison of clinical information between NCI and CIgroups at baseline

Baseline Variables	NCI group (***n*** = 857)	CI group (***n*** = 818)	P value
Gender (male, n,%)	654(76.31)	583(71.27)	0.019^*^
Average age (years, mean ± SD)	58.37 ± 10.80	62.79 ± 9.59	< 0.001^**^
Body mass index (kg/m^2^, mean ± SD)	25.08 ± 3.15	25.11 ± 3.24	0.88
Risk factors			
Diabetes (n, %)	284(33.14)	257(31.42)	0.45
Hypertension (n, %)	659(76.90)	603(73.72)	0.13
Lipid metabolism disorders (n, %)	385(44.92)	352(43.03)	0.44
Atrial fibrillation (n, %)	34(3.97)	34(4.16)	0.84
Current or previous smoking (n, %)	324(38.04)	298(36.43)	0.50
Previous mRS [scores, median (IQR)])	0.00(1.00)	0.00 (1.00)	0.24
Previous stroke (n, %)	148(17.27)	215(26.28)	< 0.0001^**^
NIHSS at baseline [scores, median (IQR)]	2.00 (2.00)	2.00 (2.00)	0.10
Neuropsychiatric symptom at 2 weeks (n, %)			
PSQI > 5	352(41.07)	384(46.94)	0.016^*^
ESS > 10	100(11.71)	92(11.27)	0.78
PHQ-9 > 9	46(5.40)	75(9.26)	0.0025^**^
GAD-7 > 9	36(4.21)	48(5.90)	0.11
Stroke subtype for TOAST (n,%)			0.02^*^
large artery atherosclerosis	169(19.72)	211(25.79)	
cardiogenic embolism	40(4.67)	41(5.01)	
small artery occlusion	266(31.04)	218(26.65)	
Other/Unknown	382(44.57)	348(42.54)	
Acute infarction type (n,%)			0.0010^*^
Single infarction	401(46.79)	338(41.32)	
Multiple infarction	294(34.31)	347(42.42)	
Simple watershed infarction	10(1.17)	18(2.20)	
No infarction	152(17.74)	115(14.06)	
Intracranial atherosclerotic stenosis (ICAS) (n,%)	154(28.62)	208(36.30)	0.006^**^
Intravenous thrombolysis (n,%)	56(6.53)	42(5.13)	0.22
Dual antiplatelet therapy (n,%)	368(49.60)	406(54.79)	0.045^*^
Secondary prevention of stroke at 2 weeks (n,%)			
Antiplatelet or anticoagulant therapy	841(98.13)	800(97.80)	0.63
Antihypertensive therapy	467(54.49)	439(53.67)	0.73
Lipid-lowering therapy	813(94.87)	786(96.09)	0.23
Hypoglycemic therapy	213(24.85)	205(25.06)	0.92

*mRS* modified Rankin Scale, *NIHSS* National Institutes of Health Stroke Scale, *PSQI* Pittsburgh Sleep Quality Index, *ESS* Epworth Sleeping Scale, *GAD-7* Anxiety Disorder-7, *PHQ-9* Patient Health Questionnaire-9. *<0.05；**<0.01

### Comparison of outcomes at 3 months between CI and NCI groups

Table 2 showed the comparison of outcomes at 3 months between CI and NCI groups. CI group have significantly worse stroke outcome (mRS ≥ 2) and SIS-16<25% than those in NCI group. After adjusted for the confounders, there were no statistic differences.

**Table 2:** Comparison of 3-months functional outcomes between CI and NCI groups

Outcome	CI group (n = 818)	NCI group (n = 857)	Unadjusted analysis		Adjusted analysis ^†^	
			Odds ratio* (95% CI)	*p* value	Odds ratio† (95% CI)	p value
mRS ≥ 2 at 3 months (n,%)	123 (15.0)	84 (9.8)	1.63(1.21-2.19)	0.0012**	1.33(0.97-1.83)	0.082
SIS-16 < Q1 at 3 months(n,%)	264 (32.27)	193 (22.52)	1.64(1.32-2.04)	< 0.0001**	1.25(0.98-1.59)	0.073

*mRS* modified Rankin Scale, *SIS-16* Stroke Impact Scale

Adjusted for gender, age, history of stroke, sleep disorders, depression, acute infarction type, TOAST type, acute infarction type, intracranial atherosclerotic stenosis and dual antiplatelet therapy at baseline

*:<0.05；**:<0.01

### Association of different change patterns of PSCI with clinical outcome at 3 months

**Fig. 2:**
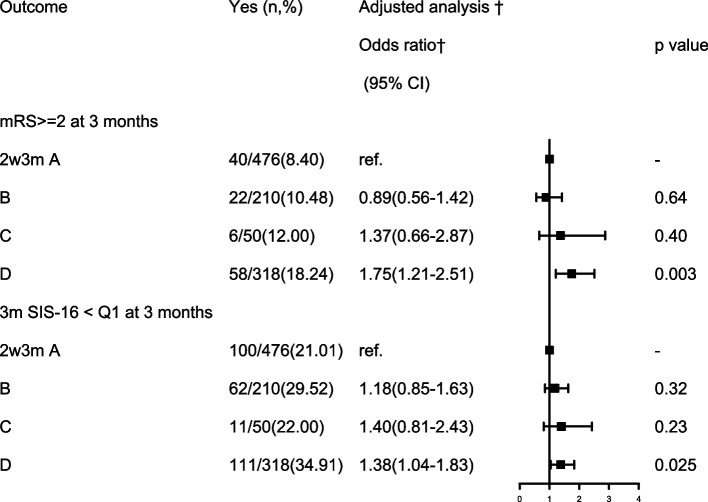
Association of different change patterns of PSCI with clinical outcome at 3 months

The association of different change patterns of PSCI with clinical outcomes after stroke at 3 months is presented in Table 3. In the univariate analysis, data showed that the persisting PSCI was associated with the adverse stroke outcomes at 3 months by higher percentage of mRS ≥ 2 and SIS-16<25% (*P* < 0.001). After adjusting for age, sex, history of stroke, combination of sleep disorders(PSQI>5), depression(PHQ-9>9), acute infarction type, stroke etiology and other potential confounding factors at baseline, patients with persisting PSCI had an increased risk of poor outcome [adjusted OR (aOR) =1.75; 95% CI, 1.21-2.51] and poor physical and social functioning[adjusted OR (aOR) =1.38; 95% CI, 1.04-1.83] at 3 months(Fig. 2). On the contrary, other 3 groups were not associated with 3-month poor outcome and physical and social functioning in this study.

**Table 3:** Comparison of 3-months functional outcomes among patients with different types of post-stroke cognitive impairment after adjusted for baseline covariates

Outcome	Yes (n,%)	Unadjusted analysis		Adjusted analysis ^†^	
		Odds ratio* (95% CI)	p value	Odds ratio† (95% CI)	p value
mRS ≥ 2 at 3 months (n,%)					
2w3 m A group	40/476(8.40)	–	–	–	–
B group	22/210(10.48)	1.01(0.65-1.56)	0.98	0.89(0.56-1.42)	0.64
C group	6/50(12.00)	1.36(0.67-2.75)	0.396	1.37(0.66-2.87)	0.40
D group	58/318(18.24)	2.17(1.56-3.02)	< 0.0001**	1.75(1.21-2.51)	0.003^**^
3 m SIS-16 < Q1 at 3 months					
2w3 m A group	100/476(21.01)	–	–	–	–
B group	62/210(29.52)	1.34(0.99-1.81)	0.05	1.18(0.85-1.63)	0.32
C group	11/50(22.00)	1.54(0.93-2.56)	0.10	1.40(0.81-2.43)	0.23
D group	111/318(34.91)	1.99(1.55-2.56)	< 0.0001**	1.38(1.04-1.83)	0.025^*^

*mRS* modified Rankin Scale, *SIS-16* Stroke Impact Scale

A group = no PSCI group: with MoCA-2w>22 and MoCA-3 m>22; B group = improved PSCI group: with MoCA-2w≦22 and MoCA-3 m>22; C group = delayed PSCI group: with MoCA-2w>22 and MoCA-3 m≦22; D group = persisting PSCI group: with MoCA-2w≦22 and MoCA-3 m≦22

Adjusted for gender, age, history of stroke, sleep disorders, depression, acute infarction type, TOAST type. Infarction type, Intracranial atherosclerotic stenosis, dual antiplatelet therapy at baseline

*:<0.05；**:<0.01

### Association of different change patterns of PSCI with clinical outcome at 1 year after adjusted for confounding factors at baseline

**Fig. 3:**
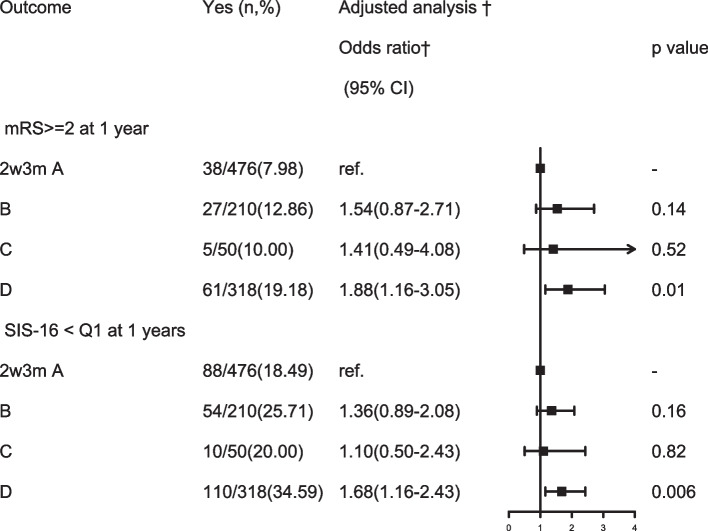
Association of different change patterns of PSCI with clinical outcome at 1 year after adjusted for confounding factors at baseline

The association of different change patterns of PSCI with clinical outcomes after stroke at 1 year is presented in Table 4. About 1054 patients had completed MoCA-2w, MoCA-3 m tests, mRS-3 m and mRS-12 m, the baseline and clinical features of the included 1054 patients are demonstrated in Supplementary File 1. Similarly, in the univariate analysis, data showed that the persisting PSCI was associated with the adverse stroke outcomes at 1 year by higher percentage of mRS ≥ 2 and SIS-16<25% (*P* < 0.001). After adjusting for age, sex, history of stroke, combination of depression (PHQ-9>9), acute infarction type, stroke etiology and other potential confounding factors at baseline, patients with persisting PSCI had an increased risk of poor outcome [adjusted OR (aOR) =1.88; 95% CI, 1.16-3.05] and poor physical and social functioning [adjusted OR (aOR) =1.68; 95% CI, 1.16-2.43] at 1 year (Fig. 3). There was no significant association between other 3 groups and 1-year poor outcome and physical and social functioning in this study.

**Table 4:** Comparison of 1-year functional outcomes among patients with different types of post-stroke cognitive impairment after adjusted for baseline covariates

Outcome	Yes (n,%)	Unadjusted analysis		Adjusted analysis ^†^	
		Odds ratio* (95% CI)	p value	Odds ratio† (95% CI)	p value
mRS ≥ 2 at 1 year					
A group	38/476(7.98)	–	–	–	–
B group	27/210(12.86)	1.70(1.01-2.87)	0.046*	1.54(0.87-2.71)	0.14
C group	5/50(10.0)	1.28(0.48-3.42)	0.62	1.41(0.49-4.08)	0.52
D group	61/318(19.18)	2.74(1.77-4.22)	< 0.0001**	1.88(1.16-3.05)	0.01^*^
SIS-16 < Q1 at 1 years					
A group	88/476(18.49)	–	–	–	–
B group	54/210(25.71)	1.53(1.04-2.25)	0.032*	1.36(0.89-2.08)	0.16
C group	10/50(20.00)	1.10(0.53-2.29)	0.79	1.10(0.50-2.43)	0.82
D group	110/318(34.59)	2.33(1.68-3.23)	< 0.0001**	1.68(1.16-2.43)	0.006^**^

*mRS* modified Rankin Scale, *SIS-16* Stroke Impact Scale

A group = no PSCI group: with MoCA-2w >22 and MoCA-3 m>22; B group = improved PSCI group: with MoCA-2w ≦22 and MoCA-3 m>22; C group = delayed PSCI group: MoCA-2w>22 and MoCA-3 m≦22; D group = persisting PSCI group: with MoCA-2w≦22 and MoCA-3 m≦22

Adjusted for gender, age, history of stroke, sleep disorders, depression, acute infarction type, TOAST type. Infarction type, Intracranial atherosclerotic stenosis, dual antiplatelet therapy at baseline

*:<0.05；**:<0.01

### Comparison of outcomes at 1 year between CI and NCI groups

There were 1054 patients with TIA/minor stroke completed MoCA-2w, MoCA-3 m tests, mRS-3 m and mRS-1y tests. According to the MoCA score, there were 368 patients had cognitive impairment (MoCA≤22) at 3 months. The CI patients have significantly worse stroke outcome (mRS ≥ 2) and SIS-16<25% than those in NCI group at 1 year (Table 5). After adjusted for the confounders at 3 months, the CI group still has dramatically higher percentage of SIS-16<25% than that in NCI group.

**Table 5:** Comparison of 1-year functional outcomes between CI and NCI groups at 3 months

Outcome	CI at 3 months (*n* = 368)	NCI at 3 months (*n* = 686)	Unadjusted analysis		Adjusted analysis ^†^	
			Odds ratio* (95% CI)	p value	Odds ratio† (95% CI)	p value
mRS ≥ 2 at 1 year	66(17.9)	65(9.48)	2.09(1.44-3.02)	< 0.0001**	1.38(0.88-2.18)	0.16
SIS-16 < Q1 at 1 year	120(32.61)	142(20.70)	1.85(1.39-2.47)	< 0.0001**	1.41(1.02-1.96)	0.040^*^

*mRS* modified Rankin Scale, *SIS-16* Stroke Impact Scale

*:<0.05；**:<0.01

### Association of different change patterns of PSCI with clinical outcome at 1 year after adjusted for confounding factors at 3 months

**Fig. 4:**
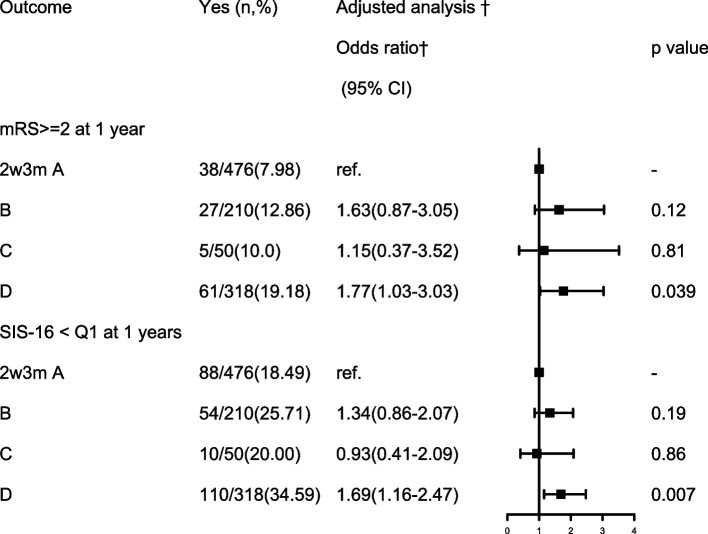
Association of different change patterns of PSCI with clinical outcome at 1 year after adjusted for confounding factors at 3 months

After adjusting for age, sex, history of stroke, combination of intracranial atherosclerotic stenosis, depression (PHQ-9>9), acute infarction type, stroke etiology and other potential confounding factors at 3 months, patients with persisting PSCI had an increased risk of poor outcome [adjusted OR (aOR) =1.77; 95% CI, 1.03-3.03] and poor physical and social functioning [adjusted OR (aOR) =1.69; 95% CI, 1.16-2.47] at 1 year (Table 6 and Fig. 4). There was no significant association between other 3 groups and 1-year poor outcome and physical and social functioning in this study.

**Table 6:** Comparison of 1-year functional outcomes among patients with different types of post-stroke cognitive impairment after adjusted for 3 months covariates

Outcome	Yes (n,%)	Unadjusted analysis		Adjusted analysis ^†^	
		Odds ratio* (95% CI)	p value	Odds ratio† (95% CI)	*p* value
mRS ≥ 2 at 1 year					
A group	38/476(7.98)	–	–	–	–
B group	27/210(12.86)	1.70(1.01-2.87)	0.046*	1.63(0.87-3.05)	0.12
C group	5/50(10.0)	1.28(0.48-3.42)	0.621	1.15(0.37-3.52)	0.81
D group	61/318(19.18)	2.74(1.77-4.22)	< 0.0001**	1.77(1.03-3.03)	0.039^*^
SIS-16 < Q1 at 1 year					
A group	88/476(18.49)	–	–		
B group	54/210(25.71)	1.53(1.03-2.25)	0.03*	1.34(0.86-2.07)	0.19
C group	10/50(20.00)	1.10(0.53-2.29)	0.79	0.93(0.41-2.09)	0.86
D group	110/318(34.59)	2.33(1.68-3.23)	< 0.0001**	1.69(1.16-2.47)	0.007^**^

*mRS* modified Rankin Scale, *SIS-16* Stroke Impact Scale

A group = no PSCI group: with MoCA-2w >22 and MoCA-3 m>22; B group = improved PSCI group: with MoCA-2w ≦22 and MoCA-3 m>22; C group = delayed PSCI group: MoCA-2w>22 and MoCA-3 m≦22; D group = persisting PSCI group: with MoCA-2w≦22 and MoCA-3 m≦22

Adjusted for gender, age, history of stroke, depression, acute infarction type, mRS score at 3 months, TOAST type, acute stroke type, intracranial atherosclerotic stenosis, dual antiplatelet therapy and lipid-lowering therapy at 3 months

*:<0.05；**:<0.01

## Discussion

In the present study, early cognitive impairment was observed in 48.84% of patients with minor stroke/TIA. Previously study reported that the rate of cognitive impairment in stroke patients varied from 21 to 70% [32]. One recent observational study reported that cognitive impairment was detected at Day 7 in 54 of 100 patients (54%) with TIA and minor stroke [8]. Another study reported that cognitive impairment (MoCA < 24) was detected within 5 days in 63% of patients with minor stroke. In the present study, cognitive impairment (MoCA≤22) was observed at 2 weeks in 48.84% of patients with TIA/minor stroke, which is lower than in previous reports. It maybe that our cohort patients were younger than those in previous studies (60 years versus 63 and 70 years in the aforementioned studies). Another reason was that we excluded those patients with pre-stroke dementia. Similar to our study, one Japanese study investigated the cognitive impairment with MoCA in 69 ischemic stroke patients (average age: 73 years), and cognitive impairment defined as a MoCA cutoff score of less than 23 was observed in 39 of 69 patients (57%) within 14 days of onset [33]. The differences might be attributed to the stroke severity, MoCA cutoff scores, and pre-existing cognitive status. The CI group had significantly higher percentage of multiple infarctions than NCI group, consistent with previous studies [34, 35]. Having multiple infarctions, compared with having a single infarction, was significantly associated with post stroke dementia [34]. Multiple brain infarctions have also been suggested to be an important risk factor for dementia. There is also a significant distribution for TOAST classifications between CI and NCI groups. Higher proportions of large-artery atherosclerosis (LAA) and cardioembolism (CE) subtypes are found in patients with CI, consistent with a previous study reporting higher prevalence of cognitive impairments in LAA and CE subtype [36]. The LAA subtype, mostly caused by the occlusion of middle cerebral artery and anterior cerebral artery, and can lead to large infarction in cerebral lobes or strategic infarctions [36–38]. LAA subtype and total anterior circulation infarction were correlated with increased PSCI risk at 3 months [36]. These pathologies play a key role in the occurrence of PSCI by causing multifocal emboli involving either bilateral or multilevel structures.

Recent hospital-based cohort studies showed that the MoCA score predicted long-term functional outcome [9]. Previous studies showed that cognitive deficits at 3 months after stroke and incident poststroke dementia to associate with poor outcome [39]. Early MoCA testing could predict 5-year functional impairment (mRS score > 2) and mortality after stroke [9]. Specially, the 14-day visuospatial/executive functions could predict 3-month functional outcomes in stroke patients with endovascular treatment [40]. Early cognitive evaluation after stroke could be affected by many factors such as delirium, tiredness and mood. Thus, using serial assessments for cognitive changes is very important. Some patients might have deteriorated while others improved, or keeping stable, respectively. According to the results and progression of cognitive assessment from 2 weeks to 3 months, we divided patients into 4 groups, and firstly explore the association between PSCI changes and functional outcomes. The present study found that the persisting PSCI type was independently associated with poor functional outcome and physical and social functioning at 3 months and 1 year, even after adjusting for age, sex, history of stroke, combination of sleep disorders, depression, acute infarction type, stroke etiology and other potential confounding factors at baseline. Moreover, the significance of the persisting PSCI to poor function outcome and physical and social functioning remained when adjusted for variables at 3 months. Extending these observations, this founding has a great clinical significance, highlighting the effectiveness of cognitive assessment with the MoCA early, as well as follow-up evaluation later. Early usage of the MoCA in different cognitive regions could predict the PSCI and future functional outcomes, which is important for screening patients with high-risk of poor prognosis and conducting an early intervention [40]. Our findings promote routine cognitive screening test and follow-up assessment after acute stroke. It is possible that in the population with minor stroke (NIHSS<3 scores), the persisting PSCI is a more sensitive predictor of functional dependence in 3-12 months. In addition, it supports the logical assumption that persisting cognitive decline at follow-up is a significant predictor for long-term functional status [41]. These results reveal the significant relationship between persisting PSCI and functional status at 3 and 12 months, even after adjusting for multiple confounding factors, emphasizing the effect of persisting PSCI on one’s ability and physical functioning independent after stroke. The findings of this study could help to identify at risk patients for cognitive decline, who will benefit from early and customized rehabilitation. Such intervention includes therapeutic lifestyle change (e.g., physical exercises, diet and sleep), cognitive training, mood and stress management [42]. The MoCA should be implemented as a crucial part of the routine follow-up clinical assessment. Its early detection of at risk stroke patients will prompt the multidisciplinary rehabilitation team to follow up with comprehensive assessment to customize care, consequently better prognosis [43].

This study shows that early cognitive screening and follow-up assessment using MoCA after stroke adds to the prediction of functional outcome up to 1 years after the event. This may partly be associated with the influence of cognitive impairment on the performance of daily activities and complexity. Previous study considered that the poor functional performance might relate to poorer adherence to treatment guidelines for PSCI patients and to have limited access to rehabilitation programs. However, in this study, we put the drug adherence into the logistic regression, still we found that the persisting PSCI still have a poor outcome at 12 months after stroke onset. It implied that the persisting PSCI patients have special pathological mechanisms from others. Hence, the persisting MoCA score ≤ 22 from baseline to 3 months might identify patients requiring special attention.

In addition, our results support the feasibility and routine use of the MoCA early after stroke. It takes about 10 minutes to rate [44], and is appropriate for stroke patients’ cognitive screening [45]. It could better reflect the underlying vascular pathology than other cognitive screening tools [46]. Besides, there are strong arguments for serial MoCA tests for cognitive follow-ups after hospital admission.

The present study had some limitations. Firstly, our study might not be fully representative of stroke in general because this study excluded patients with history of dementia and recruited patients with mild stroke. Secondly, only 24% of patients were women in the current study. Though gender was pooled into the final model, caution was needed regarding generalizability. This finding may not be generalizable to major stroke patients. Thirdly, depressive mood disorders, anxiety and delirium may have affected early cognitive performance. This condition may have an impact on functional outcome. However, serial MoCA tests were conducted at 2 weeks and 3 months, which may reduce the bias. Fourthly, we did not find statistically significantly differences in intravenous thrombolysis between CI and NCI groups. A previous study showed that intravenous thrombolysis with alteplase could improve the MMSE score at acute phase significantly than the control group [47]. However, a longitudinal study in alteplase treatment was inconsistent because the load of CMBs had been associated with cognitive impairment [48]. Thus, further studies are needed to explore the relationship between intravenous thrombolysis and PSCI. Fifthly, only a minority of patients (*n* = 10, 0.71%) used drug-treatment to improve cognition, such as donepezil (*n* = 9) and memantine(n = 1), which might influence PSCI trajectory. Therefore, the evidence of cognitive treatment was insufficient to make statistical comparisons. Additionally, this study did not collect information on physiotherapy [49] or language therapy [50], which might have an impact on cognitive changes. Further studies on relationships between treatment or rehabilitation, as well as PSCI are needed.

In conclusion, this study shows that the persisting PSCI is a strong predictor of l-year functional outcome. Patients with persisting PSCI should be given special attention. Our findings promote the use of MoCA as a routine clinical tool to identify high-risk patients in the setting of acute stroke, particularly given its brevity of administration.

## Supplementary Information

**Additional file 1.**

## Data Availability

The datasets used and/or analyzed during the current study are available from the corresponding author on reasonable request. We are unable to deposit the data in a publicly available source because there are ongoing studies using this data.
