# Correction: Persisting cognitive impairment predicts functional dependence at 1 year after stroke and transient ischemic attack: a longitudinal, cohort study

**DOI:** 10.1186/s12877-023-03763-y

**Published:** 2023-03-30

**Authors:** Xiaoling Liao, Lijun Zuo, Yanhong Dong, Yuesong Pan, Hongyi Yan, Xia Meng, Hao Li, Xingquan Zhao, Yilong Wang, Jiong Shi, Yongjun Wang

**Affiliations:** 1 Department of Neurology, Beijing Tiantan Hospital, Capital Medical University, No.119, South Fourth Ring West Road, Fengtai District, Beijing, 100070 China; 2 Alice Lee Centre for Nursing Studies, Yong Loo Lin School of Medicine, National University of Singapore, Clinical Research Centre, Block MD11, Level 2, 10 Medical Dr, Singapore, 117597 Singapore; 3 National Clinical Research Center for Neurological Diseases, Beijing Tiantan Hospital, Capital Medical University, Beijing, China

**Correction: BMC Geriatr 22, 1009 (2022)**

**https://doi.org/10.1186/s12877-022-03609-z**

After publication of this article [1], the authors reported that in the Funding section, the number belonging to the National Key Research and Development Program of China was incorrectly given as ‘2017YFC1308404' and should have read ‘2020YFC2004800’.

The original article [1] has been corrected.
