# Supplementary material for: Persisting cognitive impairment predicts functional dependence at 1 year after stroke and transient ischemic attack: a longitudinal, cohort study
Source: BMC Geriatr. 2022 Dec 31;22:1009. doi: 10.1186/s12877-022-03609-z (PMC9805269; doi:10.1186/s12877-022-03609-z)
Supplement: Supplementary file 1 — Additional file 1. [file 12877_2022_3609_MOESM1_ESM.pdf]

**Supplemental table 1 Comparison of clinical information between NCI and CI groups at baseline**

| **Baseline Variables** | **NCI group (n=686)** | **CI group (n=368)** | **P value** |
|---|---|---|---|
| Gender(male, n,%) | 530(77.26) | 253(68.75) | **0.003**** |
| Average age (years, mean±SD) | 58.85±10.67 | 62.87±9.60 | **<0.0001**** |
| Body mass index (kg/m$^2$, mean±SD) | 25.20±3.37 | 24.92±3.16 | 0.17 |
| Risk factors | | | |
| Current or previous smoking (n, %) | 258(37.61) | 137(37.23) | 0.90 |
| Diabetes (n, %) | 206(30.03) | 121(32.88) | 0.34 |
| Hypertension (n, %) | 511(74.49) | 280(76.09) | 0.57 |
| Lipid metabolism disorders (n, %) | 342(49.85) | 164(44.57) | 0.10 |
| Atrial fibrillation (n, %) | 25(3.64) | 18(4.89) | 0.33 |
| Previous stroke (n, %) | 129(18.80) | 90(24.46) | **0.03*** |
| mRS at 3 months [scores, median (IQR)]) | 1.00(1.00) | 1.00(1.00) | **0.0005**** |
| Neuropsychiatric symptom at 3 months(n, %) | | | |
| PSQI > 5 | 263(38.34) | 160(43.48) | 0.10 |
| ESS >10 | 77(11.24) | 33(8.99) | 0.26 |
| PHQ-9 scale >9 | 31(4.53) | 31(8.49) | **0.01*** |
| GAD-7 scale >9 | 19(2.77) | 17(4.64) | 0.11 |
| Stroke subtype for TOAST (n, %) | | | **0.005**** |
| large artery atherosclerosis | 139(20.26) | 97(26.36) | |
| cardiogenic embolism | 31(4.52) | 21(5.71) | |
| small artery occlusion | 208(30.32) | 95(25.82) | |
| Other/Unknown | 308(44.90) | 155(42.12) | |
| Acute infarction type(n, %) | | | **0.03*** |
| Single infarction | 321(46.79) | 160(43.48) | |
| Multiple infarction | 248(36.15) | 164(44.57) | |
| Simple watershed infarction | 14(2.04) | 5(1.36) | |
| No infarction | 103(15.01) | 39(10.60) | |
| Intracranial atherosclerotic stenosis (ICAS) (n,%) | 133(29.36) | 101(37.55) | **0.04*** |
| Intravenous thrombolysis (n,%) | 30(4.37) | 20(5.43) | 0.44 |
| Dual antiplatelet therapy (n,%) | 284(47.73) | 168(50.30) | 0.45 |
| Secondary prevention of stroke at 3 months (n,%) | | | |

| | | | |
|---|---|---|---|
| Antiplatelet or anticoagulant therapy | 626(91.25) | 338(91.85) | 0.74 |
| Antihypertensive therapy | 376(54.89) | 204(55.43) | 0.87 |
| Lipid-lowering therapy | 539(78.69) | 309(83.97) | **0.04**** |
| Hypoglycemic therapy | 152(22.19) | 87(23.64) | 0.59 |

mRS= modified Rankin Scale;NIHSS= National Institutes of Health Stroke Scale;PSQI= Pittsburgh Sleep Quality Index;ESS= Epworth Sleeping Scale; GAD-7=Anxiety Disorder-7;PHQ-9=Patient Health Questionnaire-9. *:＜0.05； **:＜0.01.
